# Management of Chronic and Gestational Hypertension of Pregnancy: A Guide for Primary Care Nurse Practitioners

**DOI:** 10.2174/1874434601812010180

**Published:** 2018-08-31

**Authors:** Leah Spiro, Donna Scemons

**Affiliations:** California State University, Los Angeles, USA

**Keywords:** Obstetics, Gastational hypertension, Pregestational hypertension, ACOG, Hypertension, Women

## Abstract

**Aim:**

The aim of this discussion paper is to outline the guidelines, according to the American Congress of Obstetricians and Gynecologists, about how to manage hypertension before and during pregnancy. Primary providers lack the knowledge to initiate treatment and manage hypertension in patients who are family planning or in the early stages of pregnancy before transferring care to an obstetrician, or perhaps patients who never do transfer care for lack of accessibility or funding. This paper aims to discuss how the Family Nurse Practitioner, or other primary care providers, may safely and efficiently maintain stable blood pressures in patients with hypertension before, during, and after pregnancy.

**Background and Implications for Nursing:**

Clinicians often defer gestational complications to obstetricians, however, it is crucial that there is a basic understanding of how to manage such issues. Primary practitioners do care for these patients during pre-gestation or fertility planning and oftentimes even during pregnancy in underserved communities with little access to obstetrical / prenatal care.

**Design:**

Discussion paper of ACOG guidelines and recommendations regarding safe management of hypertension before, during and after pregnancy.

**Data Sources:**

Inclusion criteria utilized most current research within the past 5 years, barring one source from the American Heart Association (no more current data) from 2011. This included an examination of current standards of care regarding hypertension during and before pregnancy according to the ACOG. Utilizing keywords such as hypertension, gestational hypertension, pregnancy, pharmacological management of hypertension, ACOG guidelines, treatment of hypertension in pregnancy. The decision to utilize guidelines set forth primarily by ACOG stems from ACOG being the governing body for Obstetricians / Gynecologists. Additionally, “standardization of care improves patient outcomes, which also should translate into a reduction in medical-legal exposure” [2]. As “such guidelines have been developed by specialty organizations such as the American College of Obstetricians and Gynecologists (the college),” ACOG guidelines are nationally utilized in the care of OB/GYN patients.

**Conclusion:**

It is essential for primary care clinicians to employ current research regarding hypertension surrounding pregnancy and encourage patients who are family planning to utilize all such data in order to have a healthy and successful pregnancy. In order to do so, thorough practitioners are required to care for patients throughout the spectrum of all health and wellness related situations.

## 1 INTRODUCTION

This discussion paper will present the most current data regarding the management of hypertensive disorders during pregnancy according to ACOG. Primary practitioners often lack knowledge on how to treat women who are in the process of family planning or in the early stages of pregnancy. Hypertension is an overwhelmingly common disorder among American women and lacking knowledge on how to treat hypertension in pregnancy may be detrimental to the pregnancy and health of the mother and fetus. Below is a guide to understanding the classification of hypertension in pregnancy and how to treat it.

Management of the preeclamptic patient is quite well understood, though much ambiguity seems to exist surrounding the care of the chronic or gestational hypertensive. A wide gap seems to exist in the knowledge and education of primary care providers, including Nurse Practitioners (NP). Thus, the culture has become to refer any medical issues during pregnancy to obstetricians. Safe medical practice during pregnancy adds an additional facet of complexity to the already complicated healthcare system in order to ensure the wellbeing of both the mother and fetus [1]. While caring for patients who are attempting to become pregnant, or are perhaps in the early stages of pregnancy, the primary care provider is responsible for understanding how to manage medical issues that are preexisting or may arise, especially hypertension. According to ACOG, the primary care practitioners must have a basic understanding of the management of hypertensive disorders of pregnancy, as they impact up to 10% of pregnancies worldwide and are a leading cause of maternal morbidity and mortality [1]. The ACOG recommendation also states that “less-than-optimal care of patients with preeclampsia and other hypertensive disorders of pregnancy reportedly occurs with some frequency worldwide, contributing to maternal and perinatal injury that might have been avoidable,” therefore further encouraging providers to obtain the skill and proficiency to differentiate and manage hypertensive disorders during pregnancy (2013, pg. ix).

## 2 DIAGNOSTIC CRITERIA

Establishing a concrete differentiation between the variations of hypertensive disorders remains a necessary guide for the primary provider. According to ACOG (2013), gestational hypertension is new onset systolic blood pressure of 140 mmHg or diastolic blood pressure of 90 mmHg after 20 weeks of gestation (or before 12 weeks postpartum) and without proteinuria or evidence of end-organ dysfunction, such as thrombocytopenia, increased creatinine above 1.1 mg/dL or elevated liver enzymes [1]. Gestational or chronic hypertension is considered severe when systolic blood pressure is 160 mmHg and/or diastolic blood pressure is 110 mmHg on two consecutive occasions at least four hours apart [1]. Hypertension that is established earlier than 20 weeks is likely preexisting even if it is seemingly new onset, thus pre-gestational or chronic hypertension; the presence of proteinuria suggests preeclampsia [1].

### 3 NON-PHARMACOLOGICAL MANAGEMENT

The management of patients with gestational hypertension can safely be performed as outpatients when there is no evidence of severe hypertension or progression to preeclampsia present through weekly in-office blood pressure monitoring and urine protein excretion, as well as twice weekly at home blood pressure measurements [1].

Management of patients with chronic and gestational hypertension is similar once preeclampsia has been ruled out. Non-pharmacological interventions include the patient's activity or exercise level and diet. According to Abdul Sultan *et al.* (2013), practitioners are discouraged from placing patients on strict bed rest and encouraged to maintain normal physical activity levels, as prolonged bed rest has been shown to increase risk for venous thromboembolism, especially given the physiological hypercoagulability of pregnancy [3]. Strength training and pure isometric exercise, including lifting weights, and aerobic exercise is discouraged as it can acutely elevate blood pressure to severe levels, increasing the risk for adverse events, including stroke; much research is needed in this field as exercise has also been shown to decrease blood pressure and aid in treatment [4]. ACOG however, recommends 30 minutes of moderate exercise on most days of the week in order “to stimulate placental angiogenesis and improve maternal endothelial dysfunction (pg. 29). Surprisingly, ACOG (2013) found no evidence to suggest benefits in restricting sodium intake during pregnancy, thus they recommend not limiting intake in the prevention of preeclampsia [1].

## 4 PHARMACOLOGICAL MANAGEMENT

Pharmacological management is the mainstay of treatment and is not limited to antihypertensive agents. The American Heart Association (2011) recommends low dose aspirin of 81mg or less to be initiated before 20 weeks of gestation to prevent preeclampsia as a sequelae of hypertension [1, 5]. Cochrane meta-analyses show efficacy only if initiated earlier than 20 weeks of gestation, thus aspirin is used primarily in the treatment of chronic hypertension rather than gestational hypertension or preeclampsia [1]. ACOG additionally advises the use of antiplatelet agents in women with a high baseline risk for the development of preeclampsia. Though it has a modest effect, the cost and risk is low, thus primary prevention with aspirin is recommended [1].

The primary care provider is responsible to assess women before becoming pregnant, or early in pregnancy for factors that may indicate secondary hypertension as per the *Seventh Report of the Joint National Committee on Prevention, Detection, Evaluation, and Treatment of High Blood Pressure* and establish baseline serology as a point of comparison should preeclampsia be suspected later in pregnancy (NIH, 2004). There is little data that indicates the precise target blood pressure in pregnancy [6], though much research exists in non-pregnant patients recommending pharmacological treatment for blood pressures that signify hypertension: above 160 mmHg systolic or 105 mmHg diastolic, though aggressive blood pressure lowering (below 140 mmHg systolic or 80 mmHg diastolic) is discouraged due to a likely decrease in uteroplacental blood flow from overmedication and induced hypotension [1].

All antihypertensive medications cross the placental barrier to the fetus, thus selection of antihypertensive medications is crucial to ensure minimal unfavorable effects [7]. Untreated chronic hypertension increases the risk for congenital cardiac malformation [7]. Most patients with chronic hypertension are treated with angiotensin converting enzyme inhibitors (ACE inhibitors) or Angiotensin II Receptor Blockers (ARBs), or direct renin inhibitors, all of which are associated with notable fetal renal abnormalities, oligohydramnios, pulmonary hypoplasia, fetal growth restrictions and calvarial abnormalities when taken during pregnancy [1]. Thus, according to ACOG (2013) the initiation of these medications should be avoided, and women planning pregnancy should be switched to safer medications [1]. Several oral agents have been studied in a mixed group of hypertensive patients for safety and efficacy in pregnancy in the outpatient setting including Methyldopa, Labetalol and Procardia [1].

Methyldopa, a centrally acting alpha-2 adrenergic agonist, is oftentimes used as a first line agent, mainly because of its longstanding history of safety and use in pregnancy; because of the indirect mechanism of action, blood pressure control is gradual over 6-8 hours and is best for treatment of mild hypertension rather than moderate or severe [1]. Labetalol, a nonselective beta-blocker, is often used to treat hypertension in pregnancy, though it can cause bronchospasm and should be avoided in asthmatics [1]. Labetalol has been used widely in pregnancy and has proven effective in the treatment of mild to moderate hypertension, though some data shows a slight increase in small for gestational age (SGA) infants [1]. Procardia, a calcium channel blocker, is also used often in pregnancy to treat mild to moderate hypertension and has shown an indication of adverse perinatal outcomes or decreased uterine blood flow [1]. Diuretics have some usefulness in pregnancy, specifically with salt-sensitive hypertension and for patients with reduced renal function, though some concern exists regarding the safety; careful attention must be paid to avoid hypokalemia and fetal growth restriction from intravascular volume depletion [1]. ACOG recommends Methyldopa, Labetalol and Procardia as first line medications in the treatment of hypertension in pregnancy, and the use of diuretics only as a second line medication in situations that specifically call for such a mechanism of action [1].

### 5 INDICATIONS FOR PRETERM DELIVERY AND INTRAPARTUM MANAGEMENT

Preterm delivery is not indicated for patients without complications associated with hypertension per ACOG, though each case should be analyzed on an individualized basis [1]. The recommendations for delivery are as follows: 38-39 6/7 weeks of gestation for women not requiring medication, 37- 39 6/7 weeks of gestation for women with hypertension controlled with medication, 36-37 6/7 weeks of gestation for women with severe hypertension difficult to control [1]. Patients with uncomplicated hypertension have similar outcomes to comparable patients without hypertension, thus eliminating the rationale for early delivery, optimizing outcomes for the fetus and the patient [1]. A plethora of research exists regarding the management of intrapartum hypertensive patients, though it is outside of the scope of the primary care provider and includes intravenous medications for acute blood pressure treatment, intravenous magnesium sulfate administration for seizure prophylaxis with suspected preeclampsia and serial serology. Postpartum hypertension until 12 weeks postpartum should be managed similarly, taking into account medications that are safe for breastfeeding if the mother so chooses.

## CONCLUSION

The management of hypertension in pregnancy is straightforward and can be initiated by the primary care provider while women of childbearing age are attempting to conceive and in the very early stages of pregnancy, before care has been transferred to the primary obstetrician. It is vital that providers are able to differentiate between types of hypertension in pregnancy, institute initial treatment, and manage hypertension safely and effectively throughout pregnancy.
